# U-Shaped Association between Sleep Duration, C-Reactive Protein, and Uric Acid in Korean Women

**DOI:** 10.3390/ijerph17082657

**Published:** 2020-04-13

**Authors:** Yea-Chan Lee, Da-Hye Son, Yu-Jin Kwon

**Affiliations:** 1 Department of Family Medicine, Yonsei University College of Medicine, 50-1, Yonsei-ro, Seodaemun-gu, Seoul 03722, Korea; yeachanlee92@yuhs.ac (Y.-C.L.); sonda@yuhs.ac (D.-H.S.); 2 Department of Family Medicine, Yongin Severance Hospital, Yonsei University College of Medicine, 363, Dongbaekjukjeon-daero, Giheung-gu, Yongin-si 16995, Gyeonggi-do, Korea

**Keywords:** sleep duration, high-sensitivity C-reactive protein, uric acid, inflammation

## Abstract

Serum high-sensitivity C-reactive protein (hsCRP) and serum uric acid (SUA) are biomarkers that predict chronic inflammation and cardiovascular dysfunction. Therefore, we aimed to investigate the association between sleep duration, hsCRP, and SUA in Korean women. Cross-sectional data from the Seventh Korea National Health and Nutrition Examination Survey was analyzed. The odds ratio (OR) and 95% confidence intervals (CIs) for an association between higher hsCRP (>2.0 mg/L) or higher SUA (>5.6 mg/dL) and sleep duration were calculated using multiple logistic regression analyses after adjusting for potential confounders. In total, 6151 women were included in the analysis. There was a U-shaped relationship between continuous sleep duration, hsCRP, and SUA. Compared to those who slept for 7–8 h, the ORs (95% CIs) for higher hsCRP were 1.43 (0.95–2.16) in short sleepers and 1.64 (1.09–2.48) in long sleepers after adjusting for confounders. Compared with those who slept for 7–8 h, the ORs (95% CIs) for higher SUA were 1.54 (1.04–2.26) in short sleepers and 1.94 (1.27–2.96) in long sleepers after adjusting for confounders. We found a U-shaped association between sleep duration, hsCRP, and SUA in Korean women. 7–8 h sleep was associated with lower level of hsCRP and SUA in Korean women.

## 1. Introduction

Sleep plays a vital role in human health and well-being [1,2]. Various sleep related components, such as alertness, timing of sleep, sleep efficiency, and sleep duration, are involved in systemic processes and systems, such as metabolism and the cardiovascular system [1]. Sleep duration is one of the most important components of sleep and it is easily quantifiable. Many studies have shown that too little and too much sleep are associated with diabetes, cerebrovascular diseases, cardiovascular diseases, and all-cause mortality [3,4,5]. One potential pathway linking sleep duration and cardiometabolic health is systemic inflammation.

C-reactive protein (CRP) is a stable inflammation marker and predictor of cardiovascular diseases [6]. CRP is secreted by the liver in response to various inflammatory cytokines, such as interleukin-6 (IL-6) [7]. Recently, a high-sensitivity C-reactive protein (hsCRP) test that can detect CRP more accurately and at lower concentrations has been developed and widely used due to its high predictive value [8]. Previous studies have established that elevated hsCRP is associated with cardiovascular diseases, diabetes, and mortality [9,10].

Serum uric acid (SUA) is the final product of both intracellular and extracellular purine metabolism, and requires xanthine-oxidoreductase enzyme activity [11]. SUA has both pro-oxidant and anti-oxidant activity. Accumulation of reactive oxygen species with SUA production can lead to predominantly pro-oxidant and pro-inflammatory effects [11,12,13]. SUA also induces the expression of hepatic inflammatory molecules by activating the nuclear factor kappa-light-chain-enhancer of activated B cells (NF-kB) signaling cascade [13]. Many epidemiologic studies have noted that higher SUA is significantly associated with the risk of hypertension [14,15], heart failure [16], and mortality due to cardiovascular events [17]. Further, several studies have reported that higher SUA is strongly associated with coronary atherosclerosis, left ventricular hypertrophy, and mortality due to cardiovascular events especially in women [17,18,19].

Although previous studies have investigated the association between sleep duration, hsCRP, and SUA, the results were inconsistent [20,21,22,23,24]. Some studies found that short sleep duration was associated with elevated CRP [20,21]. Richardson et al. [22] showed that short sleep duration was significantly associated with elevated CRP only in men not in women. Grandner et al. [23] found that both short and long sleep duration were associated with elevated CRP; however, these associations varied according to sex and race/ethnicity. A recent study reported that sleep duration was inversely associated with SUA in an elderly Mediterranean population with metabolic syndrome or obesity [24].

Considering the importance of sleep duration and its impact on chronic inflammation, large population-based studies are needed to investigate the association between sleep duration, hsCRP, and SUA. Therefore, we aimed to investigate the association between sleep duration, hsCRP, and SUA in Korean women.

## 2. Materials and Methods

### 2.1. Study Design and Population

We used data from the 2016–2017 Korean National Health and Nutrition Examination Survey (KNHANES) VII performed by the Korea Centers for Disease Control and Prevention (KCDC). Detailed information about KNHANES is described on the website (https://knhanes.cdc.go.kr) and previous study [25].

**Figure 1:**
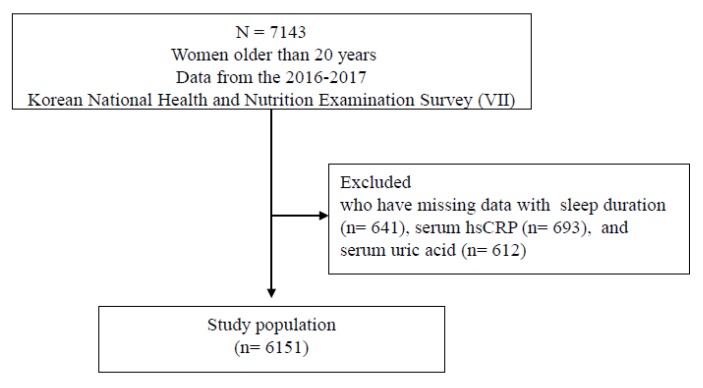
Study population selection process.

There were 7143 female participants older than 20 years in KNHANES VII. We excluded 641 participants who had missing data regarding sleep duration, 693 participants with missing data regarding hsCRP, and 612 participants with missing data regardingSUA. Finally, the data from 6151 women were included the analysis (Figure 1). Informed consent was obtained from all participants during the survey by KCDC. The Institutional Review Board of Yongin Severance Hospital approved this study (IRB No 9-2019-0008).

### 2.2. Covariates

Body mass index (BMI) was calculated as body weight (kg) divided by the square of height (m). Blood pressure was measured using a Baumanometer Wall Unit 33(0850) in the sitting position after 5 min of rest. Systolic blood pressure (SBP) and diastolic blood pressure (DBP) were recorded three times and the mean value of last two measurements was recorded. Fasting glucose and total cholesterol (TC) levels were measured after an overnight fast using a Hitachi 7600-210 Chemistry Analyzer (Hitachi Co., Tokyo, Japan).

Smoking history was defined as current smokers or those who had smoked over 100 cigarettes during their lifetime. An alcohol drinker was defined as an individual who consumed alcohol more than 2 or 3 times a week. Exercise of moderate intensity for more than 150 min a week or high intensity for more than 75 min a week was defined as regular exercise. Diabetes mellitus was defined as a blood glucose level of more than 126 mg/dL, taking diabetic medication, or undergoing insulin treatment, or a diagnosis of diabetes mellitus by a physician. Hypertension was defined as SBP ≥ 140 mmHg or DBP ≥ 90 mmHg or taking antihypertensive medication. Hypercholesterolemia was defined as total cholesterol ≥ 240 mg/dL or taking cholesterol lowering medication. Cardiovascular disease was defined as a diagnosis of stroke, myocardial infarction, or angina pectoris by a physician. EuroQol-5D (EQ5D) is a well-validated instrument to evaluate the quality of life. EQ5D consists of 5 dimensions: mobility, daily activity, pain or discomfort, self-care, and anxiety or depression [26]. We assessed depressive symptoms using a patient health questionnaire (PHQ)-9 that consisted of nine questions for screening depression in primary care; the cut-off value to identify depressive symptoms was 10 [27].

### 2.3. Assessment of High-Sensitivity CRP and Uric Acid Levels

The serum level of hsCRP was measured by an immunoturbidimetry method using Cobas (Roche, Germany). In the workshop by Centers for Disease Control and American Heart Association in 2002, the cut-points of hsCRP concentrations for purposes of cardiovascular risk assessment were classified as follows; low risk (<1.0 mg/L), average risk (1.0 to 3.0 mg/L), and high risk (>3.0 mg/L) [6]. In the Jupiter trial, participants who were indicated by hsCRP levels >2 mg/L categorized to high vascular risk [28]. High hsCRP was defined as hsCRP levels > 2.0 mg/L based on the previous studies [6,28]. In an epidemiological follow-up data from National Health and Nutrition Examination Survey, deaths due to ischemic heart disease in women significantly increased when SUA levels were in the highest quartile (5.6 mg/dL) compared with the lowest quartile (4.0 mg/dL) [17]. The Princeton school district family study defined the participants who had hyperuricemia when they had above a 90th percentile of SUA level [29]. The level of SUA was measured by Uricase, a colorimetry method using a Hitachi 7600-210 automatic biochemical analyzer (Hitachi, Japan). High SUA level was defined as SUA levels exceeding the upper 90th percentile (=5.6 mg/dL) based on the previous studies [17,29].

### 2.4. Assessment of Sleep Duration

Participants were asked the following question “When do you go to bed and wake up on a weekday?” and “When do you go to bed and wake up on a weekend?” Sleep duration was calculated as follows: (sleep duration on a weekday × 5 + sleep duration on a weekend × 2)/7. The total time spent sleeping was recorded as a continuous variable (e.g., 7 h 31 min). Moreover, we classified the sleep duration into five groups as follows: <6 h, 6–7 h, 7–8 h, 8–9 h, ≥9 h.

### 2.5. Statistical Analyses

Due to the nature of the KNHANES sampling design, we analyzed the data after considering a complex sample design. Data were presented as mean ± standard errors (SEs) for continuous variables or percentage (SEs) for categorical variables. Sleep duration was categorized as follows: <6, 6–7, 7–8, 8–9, and ≥9 h. Clinical characteristics of participants were compared using a weighted analysis of variance for continuous variables or a weighted chi-square test for categorical variables. To determine the relationship between continuous sleep duration, serum hsCRP, and SUA, we developed a restricted cubic spline curve analysis, which was applied in an unadjusted model using the R package, version 3.4.4 (http://www.R-project.org). The detailed information about ‘R’ code are described in the Appendix A. To build a model, independent variables were examined in the univariate model (Appendix B). Then, variables that were closely associated with the serum hsCRP or SUA (*p* < 0.05) were imported into the final multivariate models, along with variables that indicate to be risk factors of higher hsCRP or SUA in previous studies [30,31]. Before analysis, we checked the multicollinearity between the explanatory (predictor) variables. All variance inflation factors (VIFs) were below 5. The odds ratio (OR) and 95% confidence intervals (95% CIs) were calculated using multiple logistic regression. In model 2, we adjusted for age and BMI. In model 3, we adjusted for age, BMI, smoking, alcohol intake, exercise, hypertension, diabetes mellitus, hypercholesterolemia, and cardiovascular disease. In model 4, we additionally adjusted for EQ5D and patient health questionnaire-9 (PHQ-9) results. All statistical analyses were performed using SPSS version 25.0 (SPSS version 25.0; IBM Corp., Armonk, NY, USA). All statistical tests were two-sided and the results were considered significant at *p* < 0.05.

## 3. Results

The general characteristics of the study population according to sleep duration are described in Table 1. A large proportion of women (31.5%) reported sleeping for 7–8 h. Participants who typically slept for less than 6 h were significantly older than the other sleep duration groups (*p* < 0.001). Short sleepers (<6 h) had significantly higher BMI, SBP, DBP, and fasting glucose, HbA1c, and total cholesterol levels. The prevalence of hypertension, diabetes mellitus, and hypercholesterolemia was significantly higher among short duration sleepers. The prevalence of cardiovascular disease was higher among long sleepers (≥9 h), although this was not statistically significant (*p* = 0.594). With respect to health-related behavior, long sleepers smoked more (*p* = 0.002) and exercised less (*p* = 0.001), whereas short sleepers consumed more alcohol (*p* = 0.047). Regarding quality of life, the EQ5D scores were significantly lower among short sleepers (0.93 ± 0.01) and long sleepers (0.93 ± 0.01) (*p* < 0.001). Regarding mental health, the PHQ-9 scores were significantly higher among short sleepers (4.0 ± 0.3) and long sleepers (3.7 ± 0.3) (*p* < 0.001).

**Figure 2:**
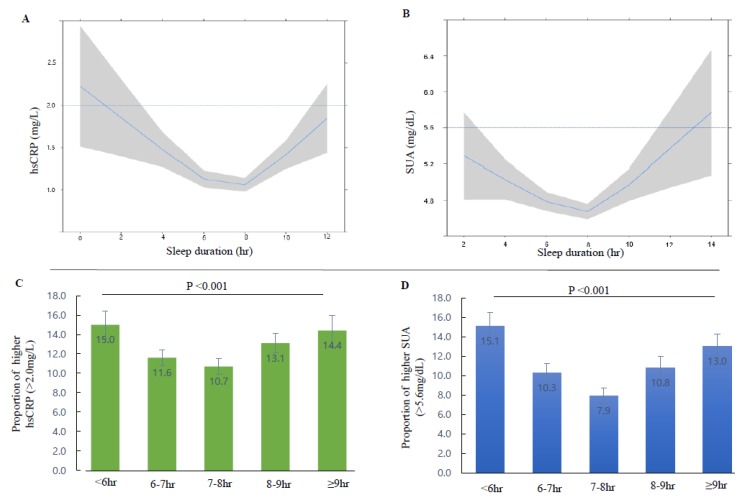
Association between serum high-sensitivity C-reactive protein (CRP), serum uric acid (SUA), and sleep duration in Korean women. (**A**) The relationship between continuous values of hsCRP and sleep duration. (**B**) The relationship between continuous values of SUA and sleep duration. (**C**) The proportion of patients with higher serum hsCRP level according to sleep duration categories. (**D**) The proportion of patients with higher SUA level according to sleep duration categories. Solid blue line (restricted cubic spline regression line) indicate predicted hsCRP and SUA by sleep duration and shaded area indicate the 95% confidence intervals.

Figure 2A,B show the U-shaped relationship of sleep duration with hsCRP and SUA levels, respectively. Figure 2C,D show the proportion of individuals with higher hsCRP (>2.0 mg/L) and SUA (>5.6 mg/dL) levels, respectively. The proportion of individuals with high hsCRP level was significantly different according to sleep duration (15.0% for <6 h, 11.6% for 6–7 h, 10.7% for 7–8 h, 13.1% for 8–9 h, and 14.4% for >9 h; *p* < 0.001). The proportion of individuals with higher SUA levels was significantly different according to sleep duration (15.1% for <6 h, 10.3% for 6–7 h, 7.9% for 7–8 h, 10.8% for 8–9 h, and 13.0% for >9 h; *p* < 0.001). U-shaped curves with the nadir in the appropriate sleep duration range (7–8 h) were seen for both hsCRP and SUA levels.

Table 2 shows the ORs and 95% CIs for higher hsCRP levels (>2.0 mg/L) according to sleep duration. Compared with those who typically slept for 7–8 h, the ORs (95% CIs) for higher hsCRP levels were 1.48 (1.11–1.97) in short sleepers and 1.41 (1.04–1.90) in long sleepers in an unadjusted model (model 1). The ORs (95% CIs) for higher hsCRP levels were 1.43 (0.95–2.16) in short sleepers and 1.64 (1.09–2.48) in long sleepers after adjusting for age, BMI, hypertension, diabetes mellitus, hypercholesterolemia, cardiovascular disease, physical activity, smoking, alcohol consumption, and EQ5D and PHQ-9 results (model 4).

Table 3 shows the ORs and 95% CIs for higher SUA levels (>5.6 mg/dL) according to sleep duration. Compared with those who typically slept for 7–8 h, the ORs (95% CIs) for higher SUA level were 2.07 (1.53–2.81) in short sleepers and 1.74 (1.27–2.39) in long sleepers in an unadjusted model (model 1). The ORs (95% CIs) for higher SUA level were 1.54 (1.04–2.26) in short sleepers and 1.94 (1.27–2.96) in long sleepers after adjusting for age, BMI, hypertension, diabetes mellitus, hypercholesterolemia, cardiovascular diseases, physical activity, smoking, alcohol consumption, and EQ5D and PHQ-9 results (model 4).

## 4. Discussion

Our study identified significant U-shaped associations between sleep duration, hsCRP, and SUA. We demonstrated that in a cohort of Korean women, individuals who sleep for <6 h or ≥9 h per day were significantly more likely to have higher levels of hsCRP and SUA compared to those who sleep for an average of 7–8 h per day. Regarding SUA, this association was still significant after adjusting for age, BMI, underlying diseases (hypertension, diabetes mellitus, hypercholesterolemia, and cardiovascular diseases), health related behavior (physical activity, smoking, and alcohol consumption), quality of life as measured by the EQ5D, and depressive symptoms as measured by the PHQ-9. After adjusting for the same confounding factors, the significant association between sleep duration and higher hsCRP was attenuated in short sleepers who slept for less than 6 h although not statistically significant.

To date, there have been inconsistent results about the relationship between sleep duration and serum hsCRP. Several previous studies have shown that a shorter sleep duration was associated with higher levels of CRP [20,21]. The longitudinal Whitehall II study [21] on London-based office employees aged 35–55 years showed that shorter sleep duration (≤ 5 h) and decreased sleep duration was significantly associated with higher levels of CRP. However, this association was diminished after adjusting for cardiometabolic risk factors such as BMI, blood pressure, cholesterol, and diabetes. A Taiwanese study [20] found that short sleepers (≤ 5.5 h) had higher levels of CRP when compared with long sleepers (>8 h). Consistent with our study, NHANES in US indicated that short and long sleep duration were associated with elevated CRP levels; however, the significant relationship between short sleep duration (<5 h) and elevated CRP levels disappeared after adjusting for medical comorbidities [23]. A meta-analysis of population-based studies showed that long sleep duration was associated with higher CRP levels, but short sleep duration was not [32]. Our results confirm these results.

Regarding SUA, few studies have investigated the relationship between sleep duration and SUA. With recent studies identifying that SUA is an important risk factor for cardiovascular diseases, more studies are needed to determine factors that affect the SUA level. Wiener et al. [33] suggested possible associations between sleep variables (daytime sleepiness, snoring, and additive composite score) and high SUA; however, they found no significant association between sleep duration and high SUA. In a recent study conducted as part of a PREDIMED-Plus trial, Papandreou et al. [24] found an inverse association between sleep duration and SUA. However, this study was limited to only elderly participants with metabolic risk factors.

The reasons for the discrepancies in these studies might be due to different categories of sleep duration, heterogeneity of study groups, and confounding variables. Our study enrolled Korean women aged over 20 years. We not only investigated the relationship between categorized sleep duration, serum hsCRP, and SUA but also the continuous relationship between sleep duration, serum hsCRP, and SUA. We also adjusted statistical models for possible confounders including metabolic risk factors, quality of life, and depressive symptoms. Our study confirmed the association between extreme (short or long) sleep duration and two markers of systemic inflammation, hsCRP, and SUA.

The mechanisms underlying the associations between sleep duration, hsCRP, and SUA are still uncertain. Short and long sleep duration may share some mechanisms involving elevated hsCRP and SUA levels.

One possible shared mechanism may involve the function of the sympathetic nerve system related to sleep deprivation or disordered sleep that leads to elevated catecholamine levels [34]. Dysregulated autonomic function can lead to changes in inflammatory gene expression due to adrenergic signaling and the subsequent production of inflammatory markers such as NF-kB or hsCRP [35]. A possible role of catecholamines in the pathogenesis of hyperuricemia has also been studied in animal models [36,37].

Several potential mechanisms may contribute to the relationship between short sleep duration and elevated hsCRP and SUA. Loss of sleep is associated with multiple hormonal changes such as reduced leptin and melatonin as well as increased cortisol and ghrelin levels [38]. These hormonal changes can lead to impaired glucose metabolism, hyperinsulinemia, obesity, hypertension, and dyslipidemia [38]. In this study, we found that short sleepers had a higher BMI, SBP, and DBP, and levels of blood glucose, HbA1c, and cholesterol. As described in many previous studies, these metabolic risk parameters are closely associated with elevated levels of hsCRP and SUA [9,10,15,16,17].

The potential mechanisms underlying the association between long sleep duration and elevated hsCRP and SUA are more speculative. First, a long sleep duration could be the result an undiagnosed illness or comorbidities that cause fatigue and lethargy [39]. Chronic inflammation is known to be involved in the main pathophysiology of many chronic diseases [40]. Although we adjusted for possible chronic diseases such as hypertension, dyslipidemia, diabetes, and cardiovascular diseases, there might be other chronic diseases, such as sleep apnea and malignancy, which were not accounted for. Second, sleeping for a prolonged period is associated with depression and an unhealthy lifestyle such as less physical activity [3]. Our study also found that both short and long sleepers have more depressive symptoms. Regarding health-related behaviors, long sleepers exercised less and smoked more. Several studies have shown that depression and inflammation are related [41,42]. The relationships between less physical activity and smoking or chronic inflammation are well established [43,44].

Our study has some limitations that need to be considered. First, owing to the cross-sectional study design, a cause-and-effect relationship could not be determined. Second, since we included only Korean women, generalization of our results to other sexes, races, and ethnicities should be done with caution. Third, we measured sleep duration using a self-reported questionnaire. The objective measurement of actual sleep duration and the consideration of other sleep variables, such as sleep quality, are needed to clarify the relationship between sleep health and inflammatory markers. Finally, there may be other residual factors that we did not adequately take into account. Despite these limitations, this study is the first large population-based study to investigate the association between sleep duration and serum hsCRP and SUA levels in Korean women.

## 5. Conclusions

Altogether, we found a U-shaped association between sleep duration, hsCRP, and SUA in Korean women. Our results indicate that 7–8 h sleep was associated with lower level of hsCRP and SUA in Korean women.

## Figures and Tables

**Table 1:** General characteristics of the study population according to sleep duration.*

	Sleep Duration					
	< 6 h	6–7 h	7–8 h	8–9 h	≥ 9 h	*p*-Value
N	840	1471	1939	1289	612	
Age	52.7 ± 0.8	48.9 ± 0.6	47.1 ± 0.5	46.4 ± 0.6	48.7 ± 1.1	<0.001
BMI (kg/m^2^)	24.3 ± 0.2	23.4 ± 0.1	23.3 ± 0.1	23.1 ± 0.1	23.3 ± 0.2	<0.001
SBP (mmHg)	118.0 ± 0.6	115.9 ± 0.6	114.4 ± 0.5	113.8 ± 0.8	114.6 ± 0.7	<0.001
DBP (mmHg)	74.5 ± 0.4	74.3 ± 0.2	73.1 ± 0.3	72.7 ± 0.3	72.1 ± 0.4	<0.001
Glucose (mg/dL)	101.9 ± 1.2	97.7 ± 0.6	96.4 ± 0.6	97.0 ± 0.7	98.4 ± 0.9	<0.001
HbA1c (%)	5.8 ± 0.0	5.6 ± 0.0	5.6 ± 0.0	5.6 ± 0.0	5.6 ± 0.0	<0.001
Total cholesterol (mg/dL)	198.5 ± 1.7	196.2 ± 1.1	193.2 ± 1.1	192.8 ± 1.0	196.0 ± 1.8	0.030
hsCRP (mg/L)	1.23 ± 0.08	1.02 ± 0.05	1.02 ± 0.05	1.06 ± 0.04	1.26 ± 0.09	0.057
SUA (mg/dL)	4.53 ± 0.04	4.35 ± 0.03	4.35 ± 0.02	4.33 ± 0.04	4.42 ± 0.05	0.002
Hypertension (yes) †	32.2 (2.3)	22.9 (1.3)	21.6 (1.3)	21.0 (1.4)	25.9 (2.3)	<0.001
Diabetes (yes) †	13.9 (1.5)	9.3 (0.9)	8.0 (0.9)	9.5 (0.8)	9.8 (1.3)	0.001
Cardiovascular diseases (yes) †	2.8 (0.6)	2.8 (0.4)	3.0 (0.4)	3.2 (0.6)	4.1 (0.9)	0.594
Hypercholesterolemia (yes) †	27.1 (1.7)	24.1 (1.2)	20.6 (1.0)	19.9 (1.3)	19.8 (1.8)	0.001
Smoking (yes) †	13.0 (1.1)	9.8 (1.1)	9.4 (0.7)	8.4 (0.8)	14.1 (1.6)	0.002
Alcohol (yes) †	14.2 (1.2)	10.2 (0.8)	13.2 (0.7)	12.3 (1.0)	13.4 (1.5)	0.047
Physical activity (yes) †	44.0 (1.8)	46.3 (1.6)	45.6 (1.6)	44.1 (1.6)	33.7 (2.4)	0.001
EQ5D	0.93 ± 0.01	0.95 ± 0.00	0.95 ± 0.00	0.95 ± 0.00	0.93 ± 0.01	<0.001
PHQ-9	4.0 ± 0.3	2.8 ± 0.2	3.1 ± 0.1	3.0 ± 0.2	3.7 ± 0.3	<0.001

Abbreviations: BMI, body-mass index; SBP, systolic blood pressure; DBP, diastolic blood pressure; HbA1c, glycated hemoglobin; hsCRP, high-sensitivity C-reactive protein; SUA, serum uric acid; EQ5D, EuroQol-5D; PHQ-9, patient health questionnaire-9. † Categorical variables. * Data are presented as mean± standard errors (SEs) for continues variables or percentage (SE) for categorical variables.

**Table 2:** Odds ratio and 95% confidence intervals for higher high-sensitivity C-reactive protein (>2.0 mg/L) according to sleep duration.

	Sleep Duration				
	< 6 h	6–7 h	7–8 h	8–9 h	≥ 9 h
Model 1	1.48 (1.11–1.97)	1.10 (0.88–1.38)	Ref (1)	1.26 (0.99–1.59)	1.41 (1.04–1.90)
Model 2	1.20 (0.89–1.62)	1.09 (0.86–1.39)	Ref (1)	1.32 (1.06–1.65)	1.48 (1.10–1.99)
Model 3	1.28 (0.93–1.77)	1.14 (0.87–1.45)	Ref (1)	1.37 (1.10–1.72)	1.42 (1.04–1.93)
Model 4	1.43 (0.95–2.16)	1.19 (0.87–1.64)	Ref (1)	1.21 (0.87–1.70)	1.64 (1.09–2.48)

Model 1: unadjusted; Model 2: adjusted for age and BMI; Model 3: adjusted for age, BMI, hypertension, diabetes mellitus, hypercholesterolemia, cardiovascular diseases, physical activity, smoking, and alcohol consumption; Model 4: adjusted for age, BMI, hypertension, diabetes mellitus, hypercholesterolemia, cardiovascular diseases physical activity, smoking, alcohol consumption, and EQ5D and PHQ-9 scores.

**Table 3:** Odds ratio and 95% confidence intervals for higher serum uric acid (>5.6 mg/dL) according to sleep duration.

	Sleep Duration				
	< 6 h	6–7 h	7–8 h	8–9 h	≥ 9 h
Model 1	2.07 (1.53–2.81)	1.35 (1.02–1.78)	Ref (1)	1.42 (0.98–2.06)	1.74 (1.27–2.39)
Model 2	1.74 (1.30–2.33)	1.33 (1.01–1.75)	Ref (1)	1.49 (1.02–2.17)	1.78 (1.29–2.46)
Model 3	1.81 (1.37–2.37)	1.40 (1.05–1.86)	Ref (1)	1.60 (1.10–2.35)	1.82 (1.34–2.49)
Model 4	1.54 (1.04–2.26)	1.13 (0.79–1.62)	Ref (1)	1.35 (0.95–1.94)	1.94 (1.27–2.96)

Model 1: unadjusted; Model 2: adjusted for age and BMI; Model 3: adjusted for age, BMI, hypertension, diabetes mellitus, hypercholesterolemia, cardiovascular diseases, physical activity, smoking, and alcohol consumption; Model 4: adjusted for age, BMI, hypertension, diabetes mellitus, hypercholesterolemia, cardiovascular diseases, physical activity, smoking, alcohol consumption, and EQ5D and PHQ-9 scores.

## Appendix A

Restricted cubic spline curve analyses: R package, version 3.4.4 (http://www.R-project.org). Code install.packages(“rms”) library(rms) rawdata=read.csv(“D:/R/sleepduration.csv”) dim(rawdata) attach(rawdata) names(rawdata) HE_hsCRP_1<-rawdata$HE_hsCRP_ HE_Uacid<-rawdata$HE_Uacid sleep duration<-rawdata$ts60 f_uni <- ols(HE_hsCRP_1~ rcs(sleep duration) data=rawdata, x=TRUE) sleep duration <- seq(0,12,2) p_uni <- Predict(f_uni, sleep duration = sleep duration) plot(p_uni,xlab=‘ sleep duration ‘, ylab=‘HS CRP’) f_uni <- ols(HE_Uacid~ rcs(ts sleep duration),data=rawdata, x=TRUE) sleep duration <- seq(0,12,2) p_uni <- Predict(f_uni, sleep duration = sleep duration) plot(p_uni,xlab=‘ sleep duration’, ylab=‘Uacid’)

## Appendix B

**Table A1:** Univariate analysis: Factors associated with hsCRP and SUA.

	hsCRP			Serum Uric Acid		
	Exp (B)	95% CI	*p*-Value	Exp (B)	95% CI	*p*-Value
Age	1.009	1.003–1.015	0.006	1.015	1.007–1.022	<0.001
BMI (kg/m^2^)	1.210	1.182–1.238	<0.001	1.157	1.132–1.183	<0.001
Hypertension (yes)	0.569	0.477–0.679	<0.001	0.444	0.359–0.548	<0.001
Diabetes mellitus (yes)	0.452	0.351–0.582	<0.001	0.456	0.337–0.616	<0.001
Hypercholesterolemia (yes)	0.751	0.627–0.900	0.002	0.628	0.504–0.782	<0.001
Cardiovascular disease (yes)	0.680	0.482–0.679	0.028	0.529	0.389–0.719	<0.001
Physical activity (yes)	1.281	1.053–1.557	0.013	1.019	0.847–1.226	0.840
Smoking (yes)	0.712	0.552–0.919	0.009	0.556	0.419–0.739	<0.001
Alcohol (yes)	1.122	0.814–1.546	0.482	0.708	0.546–0.916	0.009
EQ5D	0.223	0.124–0.400	<0.001	0.213	0.109–0.416	<0.001
PHQ-9	1.018	0.988–1.048	0.238	1.034	1.001–1.069	0.045

## References

[1] Buysse D.J. (2014). Sleep health: Can we define it? Does it matter?. Sleep.

[2] Shankar A., Charumathi S., Kalidindi S. (2011). Sleep duration and self-rated health: The national health interview survey 2008. Sleep.

[3] Yin J., Jin X., Shan Z., Li S., Huang H., Li P., Peng X., Peng Z., Yu K., Bao W. (2017). Relationship of sleep duration with all-cause mortality and cardiovascular events: A systematic review and dose-response meta-analysis of prospective cohort studies. J. Am. Heart Assoc..

[4] Li W., Wang D., Cao S., Yin X., Gong Y., Gan Y., Zhou Y., Lu Z. (2016). Sleep duration and risk of stroke events and stroke mortality: A systematic review and meta-analysis of prospective cohort studies. Int. J. Cardiol..

[5] Shan Z., Ma H., Xie M., Yan P., Guo Y., Bao W., Rong Y., Jackson C.L., Hu F.B., Liu L. (2015). Sleep duration and risk of type 2 diabetes: A meta-analysis of prospective studies. Diabetes Care.

[6] Pearson T.A., Mensah G.A., Alexander R.W., Anderson J.L., Cannon R.O., Criqui M., Fadl Y.Y., Fortmann S.P., Hong Y., Myers G.L. (2003). Markers of inflammation and cardiovascular disease: Application to clinical and public health practice: A statement for healthcare professionals from the centers for disease control and prevention and the american heart association. Circulation.

[7] Castell J.V., Gómez-lechón M.J., David M., Fabra R., Trullenque R., Heinrich P.C. (1990). Acute-phase response of human hepatocytes: Regulation of acute-phase protein synthesis by interleukin-6. Hepatology.

[8] Rifai N., Tracy R.P., Ridker P.M. (1999). Clinical efficacy of an automated high-sensitivity c-reactive protein assay. Clin. Chem..

[9] Parrinello C.M., Lutsey P.L., Ballantyne C.M., Folsom A.R., Pankow J.S., Selvin E. (2015). Six-year change in high-sensitivity c-reactive protein and risk of diabetes, cardiovascular disease, and mortality. Am. Heart J..

[10] Wang A., Liu J., Li C., Gao J., Li X., Chen S., Wu S., Ding H., Fan H., Hou S. (2017). Cumulative exposure to high-sensitivity c-reactive protein predicts the risk of cardiovascular disease. J. Am. Heart Assoc..

[11] Maiuolo J., Oppedisano F., Gratteri S., Muscoli C., Mollace V. (2016). Regulation of uric acid metabolism and excretion. Int. J. Cardiol..

[12] Kang D.H., Ha S.K. (2014). Uric acid puzzle: Dual role as anti-oxidantand pro-oxidant. Electrolyte Blood Press. Ebp..

[13] Spiga R., Marini M.A., Mancuso E., Di Fatta C., Fuoco A., Perticone F., Andreozzi F., Mannino G.C., Sesti G. (2017). Uric acid is associated with inflammatory biomarkers and induces inflammation via activating the nf-kappab signaling pathway in hepg2 cells. Arterioscler. Thromb. Vasc. Biol..

[14] Alper A.B., Chen W., Yau L., Srinivasan S.R., Berenson G.S., Hamm L.L. (2005). Childhood uric acid predicts adult blood pressure: The bogalusa heart study. Hypertension.

[15] Mellen P.B., Bleyer A.J., Erlinger T.P., Evans G.W., Nieto F.J., Wagenknecht L.E., Wofford M.R., Herrington D.M. (2006). Serum uric acid predicts incident hypertension in a biethnic cohort: The atherosclerosis risk in communities study. Hypertension.

[16] Ekundayo O.J., Dell’Italia L.J., Sanders P.W., Arnett D., Aban I., Love T.E., Filippatos G., Anker S.D., Lloyd-Jones D.M., Bakris G. (2010). Association between hyperuricemia and incident heart failure among older adults: A propensity-matched study. Int. J. Cardiol..

[17] Fang J., Alderman M.H. (2000). Serum uric acid and cardiovascular mortality the nhanes i epidemiologic follow-up study, 1971–1992. National health and nutrition examination survey. JAMA.

[18] Tuttle K.R., Short R.A., Johnson R.J. (2001). Sex differences in uric acid and risk factors for coronary artery disease. Am. J. Cardiol..

[19] Matsumura K., Ohtsubo T., Oniki H., Fujii K., Iida M. (2006). Gender-related association of serum uric acid and left ventricular hypertrophy in hypertension. Circ. J. Off. J. Jpn. Circ. Soc..

[20] Chiang J.K. (2014). Short duration of sleep is associated with elevated high-sensitivity c-reactive protein level in taiwanese adults: A cross-sectional study. J. Clin. Sleep Med. Off. Publ. Am. Acad. Sleep Med..

[21] Ferrie J.E., Kivimaki M., Akbaraly T.N., Singh-Manoux A., Miller M.A., Gimeno D., Kumari M., Davey Smith G., Shipley M.J. (2013). Associations between change in sleep duration and inflammation: Findings on c-reactive protein and interleukin 6 in the whitehall ii study. Am. J. Epidemiol..

[22] Richardson M.R., Churilla J.R. (2017). Sleep duration and c-reactive protein in us adults. South. Med. J..

[23] Grandner M.A., Buxton O.M., Jackson N., Sands-Lincoln M., Pandey A., Jean-Louis G. (2013). Extreme sleep durations and increased c-reactive protein: Effects of sex and ethnoracial group. Sleep.

[24] Papandreou C., Babio N., Diaz-Lopez A., Martinez-Gonzalez M.A., Becerra-Tomas N., Corella D., Schroder H., Romaguera D., Vioque J., Alonso-Gomez A.M. (2019). Sleep duration is inversely associated with serum uric acid concentrations and uric acid to creatinine ratio in an elderly mediterranean population at high cardiovascular risk. Nutrients.

[25] Kweon S., Kim Y., Jang M.-J., Kim Y., Kim K., Choi S., Chun C., Khang Y.-H., Oh K. (2014). Data resource profile: The korea national health and nutrition examination survey (knhanes). Int. J. Epidemiol..

[26] Devlin N.J., Brooks R. (2017). Eq-5d and the euroqol group: Past, present and future. Appl. Health Econ. Health Policy..

[27] Levis B., Benedetti A., Thombs B.D. (2019). Accuracy of patient health questionnaire-9 (phq-9) for screening to detect major depression: Individual participant data meta-analysis. BMJ.

[28] Ridker P.M. (2003). Rosuvastatin in the primary prevention of cardiovascular disease among patients with low levels of low-density lipoprotein cholesterol and elevated high-sensitivity c-reactive protein: Rationale and design of the jupiter trial. Circulation.

[29] Laskarzewski P.M., Khoury P., Morrison J.A., Kelly K., Glueck C.J. (1983). Familial hyper- and hypouricemias in random and hyperlipidemic recall cohorts: The princeton school district family study. Metab. Clin. Exp..

[30] Jung Y.E., Kang K.Y. (2019). Elevated hs-crp level is associated with depression in younger adults: Results from the korean national health and nutrition examination survey (knhanes 2016). Psychoneuroendocrinology.

[31] Stewart S.H., Mainous A.G., Gilbert G. (2002). Relation between alcohol consumption and c-reactive protein levels in the adult us population. J. Am. Board Fam. Pract..

[32] Irwin M.R., Olmstead R., Carroll J.E. (2016). Sleep disturbance, sleep duration, and inflammation: A systematic review and meta-analysis of cohort studies and experimental sleep deprivation. Biol. Psychiatry..

[33] Wiener R.C., Shankar A. (2012). Association between serum uric acid levels and sleep variables: Results from the national health and nutrition survey 2005–2008. Int. J. Inflamm..

[34] Irwin M., Thompson J., Miller C., Gillin J.C., Ziegler M. (1999). Effects of sleep and sleep deprivation on catecholamine and interleukin-2 levels in humans: Clinical implications. J. Clin. Endocrinol. Metab..

[35] Irwin M.R., Cole S.W. (2011). Reciprocal regulation of the neural and innate immune systems. Nat. Rev. Immunol..

[36] Sumi T., Umeda Y. (1977). Adrenergic regulation of the plasma levels of purine metabolites in the rat. Eur. J. Pharmacol..

[37] Yonetani Y., Ishii M., Ogawa Y. (1979). Stimulation by catecholamine of purine catabolism in rats and chickens. Jpn. J. Pharmacol..

[38] Leproult R., Van Cauter E. (2010). Role of sleep and sleep loss in hormonal release and metabolism. Endocr. Dev..

[39] Wen Y., Pi F.H., Guo P., Dong W.Y., Xie Y.Q., Wang X.Y., Xia F.F., Pang S.J., Wu Y.C., Wang Y.Y. (2016). Sleep duration, daytime napping, markers of obstructive sleep apnea and stroke in a population of southern china. Sci. Rep..

[40] Zhong J., Shi G. (2019). Editorial: Regulation of inflammation in chronic disease. Front. Immunol..

[41] Matthews K.A., Schott L.L., Bromberger J.T., Cyranowski J.M., Everson-Rose S.A., Sowers M. (2010). Are there bi-directional associations between depressive symptoms and c-reactive protein in mid-life women?. Brain Behav. Immun..

[42] Tayefi M., Shafiee M., Kazemi-Bajestani S.M.R., Esmaeili H., Darroudi S., Khakpouri S., Mohammadi M., Ghaneifar Z., Azarpajouh M.R., Moohebati M. (2017). Depression and anxiety both associate with serum level of hs-crp: A gender-stratified analysis in a population-based study. Psychoneuroendocrinology.

[43] Plaisance E.P., Grandjean P.W. (2006). Physical activity and high-sensitivity c-reactive protein. Sports Med..

[44] LaMonte M.J., Durstine J.L., Yanowitz F.G., Lim T., DuBose K.D., Davis P., Ainsworth B.E. (2002). Cardiorespiratory fitness and c-reactive protein among a tri-ethnic sample of women. Circulation.

